# Association between Polymorphisms in CFH, ARMS2, CFI, and C3 Genes and Response to Anti-VEGF Treatment in Neovascular Age-Related Macular Degeneration

**DOI:** 10.3390/biomedicines10071658

**Published:** 2022-07-10

**Authors:** Oyuna S. Kozhevnikova, Anzhella Zh. Fursova, Anna S. Derbeneva, Ida F. Nikulich, Mikhail S. Tarasov, Vasiliy A. Devyatkin, Yulia V. Rumyantseva, Darya V. Telegina, Nataliya G. Kolosova

**Affiliations:** 1 Federal Research Center Institute of Cytology and Genetics SB RAS, Pr. Lavrentiev, 10, 630090 Novosibirsk, Russia; anzhellafursova@yandex.ru (A.Z.F.); anna.derbeneva93@gmail.com (A.S.D.); kalman87@mail.ru (M.S.T.); devyatkin@bionet.nsc.ru (V.A.D.); rumyantseva@bionet.nsc.ru (Y.V.R.); telegina@bionet.nsc.ru (D.V.T.); kolosova@bionet.nsc.ru (N.G.K.); 2 State Novosibirsk Regional Clinical Hospital, St. Nemirovich-Danchenko, 130, 630087 Novosibirsk, Russia; ida_2207@mail.ru; 3 Department of Ophthalmology, Novosibirsk State Medical University, Pr. Krasny, 52, 630091 Novosibirsk, Russia

**Keywords:** age-related macular degeneration, neovascularization, anti-VEGF therapy, regulatory SNP, pharmacogenomics

## Abstract

Neovascular age-related macular degeneration (nAMD) is the leading cause of vision loss in the elderly. The gold standard of nAMD treatment is intravitreal injections of vascular endothelial growth factor (VEGF) inhibitors. Genetic factors may influence the response to anti-VEGF therapy and result in a high degree of response variability. The aim of the study was to evaluate the association of the polymorphisms in genes related to the complement system (rs2285714-CFI, rs10490924-ARMS2, rs2230199-C3, rs800292-CFH, and rs6677604-CFH) with nAMD its clinical features and optical coherent tomography (OCT) biomarkers of treatment response to anti-VEGF therapy. Genotyping by allele-specific PCR was performed in 193 AMD patients and 147 age-matched controls. A prospective study of the dynamics of changes in OCT biomarkers during aflibercept treatment included 110 treatment-naive patients. Allele T rs10490924 was associated with the increased risk of nAMD. For both rs800292 and rs6677604, carriage of the A allele was protective and decreased the nAMD risk. Associations of rs2230199 with central retinal thickness (CRT) and intraretinal cysts were revealed. The height of pigment epithelium detachment and the height of neuroretinal detachment were significantly higher in carriers of the minor allele of rs2285714, both at baseline and during treatment. The reduction of CRT was associated with higher CRT at baseline and the presence of the T allele of rs2285714. By the end of one-year follow-up the patients homozygous for the minor allele rs2285714 had significantly higher odds of the presence of anastomoses and loops and active neovascular membrane. Furthermore, minor allele carriers had decreased levels of complement factor I level in aqueous humor but not in the plasma, which may be due to the influence of rs2285714 on tissue-specific splicing. Our results suggest that the severity of AMD macular lesions is associated with rs2285714 and rs2230199 polymorphisms, which could be explained by their high regulatory potential. Patients with the minor allele of rs2285714 respond worse to antiangiogenic therapy.

## 1. Introduction

Age-related macular degeneration (AMD) is a progressive complex multifactorial disease that develops in patients over 50 years of age, which leads to a decrease in central vision and central blindness [1]. AMD is caused by a combination of risk factors, which together define an individual’s predisposition to AMD, and include ageing, environmental and lifestyle risk factors, and genetic predisposition [2]. As the global population ages, the incidence of AMD is projected to increase.

In early stages, AMD is saliently characterized by the accumulation of drusen, which are extracellular deposits underneath the retinal pigment epithelium (RPE). During the course of the disease, drusen increase in number and size, and AMD can progress into advanced stages in which vision loss occurs [3]. Clinically, AMD is divided into neovascular (nAMD), or wet, and dry forms. Although only 20% of patients with AMD are diagnosed with wet AMD, nAMD accounts for 90% cases of severe vision loss [4]. The nAMD is characterized by macular neovascularization (MNV)—an invasion of vascular and associated tissues into the outer retina, subretinal space, or sub-retinal pigment epithelium (sub-RPE) space in varying combinations, leading to the accumulation of subretinal fluid (SRF) and intraretinal fluid (IRF) and RPE detachment (PED) [1]. The current classification of MNVs divides them according to their localization into type 1 (grown from the choroid below the RPE), type 2 (grown from the choroid through RPE), and type 3 (grown from the retina toward the RPE) [5]. At present, optical coherence tomography (OCT) is the most widely used technology for the diagnosis and follow-up of nAMD patients [6]. New imaging technologies—optical coherence tomography (OCT) and OCT-angiography—have an ability to detect abnormalities not imaged by previous methods and have greater precision for many entities that are imaged using these technologies [1]. OCT allows one to identify specific retinal biomarkers of nAMD disease activity and to offer personalized management of nAMD, providing comprehensive information about the patient’s visual recovery during treatment [6,7]. 

Currently, more than half of the AMD heritability can be explained by genetic variations in or near genes of the complement cascade [2]. The complement system is a major part of innate immunity and plays an essential role in cellular homeostasis, tissue remodeling, and host defense and inflammation [8]. Common and rare variants in the CFH, CFI, CFB, and C3 complement genes, as well as the complete deletion of the complement factor H-related (CFHR) genes CFHR-1 and CFHR-3, are associated with modifying an individual’s risk of developing AMD [8,9,10,11]. In addition to mutations in complement genes, a polymorphism (rs10490924) in ARMS2 shows the highest association with AMD [11]. Recent data showed that ARMS2 also mediates AMD risk by altering complement activation, similar to complement factor H [12]. Therefore, there are strong reasons to believe that the complement system plays a central role in the pathogenesis of AMD, and excessive activation of the alternative complement pathway is one of the main factors of the disease [2].

A gold standard for nAMD treatment is the intravitreal injection (IVI) of drugs targeting vascular endothelial growth factor (VEGF). The efficacy and safety of this treatment method has been confirmed by the results of many randomized clinical trials [13,14,15,16,17]. Although anti-VEGF agents have shown a dramatic breakthrough in nAMD treatment, it became apparent that the effectiveness of treatment is not always the same, and patients show an individualized response to therapy. Most patients require frequent repeated injections and long-term follow-up, causing a high burden on the healthcare system [18]. Due to the highly heritable nature of AMD, it has been hypothesized that genetic factors may influence response to therapy for AMD and that personalization of therapy may result in better outcomes [19]. Genetic markers are independent of disease duration and therefore may explain treatment outcome variability [3]. So far there has been little data acquired on predictors for the individual response to anti-VEGF treatment [20]. Such genetic factors could be population specific.

Here we analyzed polymorphisms rs2285714 (CFI), rs10490924 (ARMS2), rs2230199 (C3), rs800292 (CFH), and rs6677604 (CFH) (Table 1) for the association with nAMD in a Caucasian sample from Western Siberia. The main aim of the study was to determine the effect of gene polymorphisms related to the complement system on the dynamics of the functional and anatomical parameters of the retina according to OCT during anti-VEGF therapy. In addition, we studied the functional importance of these polymorphisms by in silico analysis, as well as the correlation of systemic and locally produced complement factor I (FI) levels with disease status and CFI genotype. We found that rs10490924 (ARMS2), rs800292 (CFH), and rs6677604 (CFH) polymorphisms are strongly associated with nAMD in Western Siberia, and the response to antiangiogenic therapy differed according to the patient’s specific rs2285714 (CFI) and rs2230199 (C3) genotype. 

## 2. Materials and Methods

### 2.1. Study Participants

This study was conducted in accordance with the ethical principles of the Declaration of Helsinki and the National Standard for Good Clinical Practice and was approved by the Institutional Review Board at the Institute of Cytology and Genetics SB RAS. All subjects gave signed informed consent. The case group consisted of 193 patients (64 men and 129 women with a mean age of 70.4 ± 8.4 years) diagnosed with nAMD at the Department of Ophthalmology of the Novosibirsk Regional Clinical Hospital. The control group comprised 147 hospital-based age-matched subjects (60 men and 87 women with a mean age of 69.0 ± 7.8 years), undergoing routine cataract surgery without a history of AMD and macular changes such as drusen or pigment abnormalities. 

A complete ophthalmological examination was performed, including visometry, biomicroscopy, ophthalmoscopy, and optical coherence tomography (OCT). The criterion for inclusion in the case group was the presence of AMD with macular neovascularization (MNV). Exclusion criteria from the study were active neovascularization in the periphery of the retina and in the anterior segment of the eye, a history of laser photocoagulation, medical intravitreal therapy in history, spherical equivalent more than ±6.0 diopters, uveitis, geographic atrophy, surgical interventions on the vitreous body, the presence of signs of intraocular inflammation, pathology of the vitreomacular interface with traction component, polypoidal choroidal vasculopathy, or any other confounding retinopathies. 

### 2.2. OCT Subgroup Prospective Study

This sub-study included 110 treatment-naive patients (115 eyes) diagnosed with nAMD. After being diagnosed, all patients began to receive anti-VEGF therapy. Intravitreal injections (IVI) of aflibercept (Regeneron, Leverkusen, Germany) 0.05 mL (2 mg) were administered according to the standard method in the operating room after local epibulbar anesthesia with an alkaine solution (Alcon, Fort Worth, TX, USA) through a 27 G needle at least 3 mm from the limbus. Three successive injections were performed with an interval of 4 weeks, and subsequent therapy was carried out in an individualized regimen (Treat and Extend); the required number of injections was determined depending on the morphological response according to OCT data. The follow-up was 13 months.

To assess the effectiveness of therapy, a clinical and instrumental examination of patients was performed by OCT, OCT-angiography (Cirrus HD-OCT, Humphrey Zeiss, Inc., Wetzlar, Germany), and visometry, with the determination of best corrected visual acuity (BCVA) at baseline, after 3-IVI, 5-IVI, and final IVI. The following parameters were assessed: angiographic type of MNV, BCVA, central retinal thickness (CRT), height of pigment epithelium detachment (PED), height of neuroretinal detachment with subretinal fluid (SRF), the presence of intraretinal fluid (IRF), the presence of intraretinal cysts (IRC), activity of MNV, and presence of anastomoses and loops. BCVA was estimated using a letter count on the Early Treatment of Diabetic Retinopathy Study (ETDRS) chart.

### 2.3. Sample Collection

The patients were informed and provided written consent to the collection and scientific use of the specimen prior to the procedure. Fasting peripheral blood samples were collected in vacutainers with EDTA between 8:00 and 9:00 a.m. Samples were centrifuged for 15 min at 2000× *g* at 4 °C to obtain the plasma. Aqueous humor samples were obtained from 24 AMD patients before intravitreal injection of anti-VEGF and 22 age-matched controls during cataract surgery in a volume of approximately 50 μL per sample. All samples were stored at −70 °C until further analysis.

### 2.4. DNA Isolation and Genotyping

Genomic DNA was isolated from venous blood using a QIAamp DNA Blood Mini Kit (Qiagen, Hilden, Germany) according to the manufacturer’s protocol. Genotyping was carried out by TaqMan-based allelic discrimination assays. LNA (locked nucleic acid) modifications were used to obtain the optimal melting temperature in probes. Primers and probes were designed using Primer-Blast (https://www.ncbi.nlm.nih.gov/tools/primer-blast/, accessed on 1 January 2020) and Oligo Analyzer (version 1.0.3). The primer and fluorescent labeled probe sequences are shown in Table 2. PCR was performed in a 20 μL reaction volume containing 20 ng of genomic DNA, BioMaster HS-qPCR (2× buffer (Biolabmix, Novosibirsk, Russia), 0.3 mM primers, and 0.1 mM FAM/VIC-conjugated probes. PCR thermal cycling conditions were as follows: denaturation for 3 min at 95 °C followed by 35 cycles, including denaturation at 95 °C for 10 s; and primer annealing and subsequent elongation at 60 °C for 30 s. Amplification was conducted using a CFX96 Thermal Cycler (Bio-Rad, Hercules, CA, USA). Each cycle was accompanied by the detection of a fluorescent signal in the ranges corresponding to the fluorescence intervals of the FAM and VIC labels. The PCR data were processed using Bio-Rad CFX Manager 3.1 software, Russian Edition #1845028, Novosibirsk, Russia. To ensure the accuracy of the genotyping data, a sample of each genotype was used in all PCR runs, and 10% random samples were re-genotyped with a concordance rate of 100%. To verify the results of allelic discrimination, we used Sanger sequencing in samples from different genotypes on an ABI 3500 DNA sequencer (Thermo Fisher Scientific, Waltham, MA, USA) by means of the BigDye Terminator v3.1 Cycle Sequencing Kit (Thermo Fisher Scientific, Waltham, MA, USA). Supplemental Figure S1 shows the allelic discrimination results.

### 2.5. Measurements of Complement Factor I Level

Complement factor I (FI) level was measured by ELISA (SEB978Hu, Cloud-Clone Co., Wuhan, China) in diluted plasma (54 AMD cases and 25 age-matched controls) and aqueous humor (24 AMD cases and 22 age-matched controls) following the manufacturer’s protocol.

### 2.6. In Silico Analysis of SNPs Regulatory Potential

To estimate the regulatory potential and downstream functional effects of the SNPs, we analyzed the available data of epigenetic effects (HaploReg, RegulomeDB), functional predictions (SNPinfo), and expression and alternative splicing quantitative traits (GTEx consortium atlas) by using the variant annotation database VannoPortal [21].

### 2.7. Statistical Analysis

To evaluate the effects of the SNPs, odds ratio (OR) and 95% confidence interval (CI) were calculated by logistic regression analysis adopting codominant, dominant, recessive, and additive models of inheritance using SNPstats [22]. To choose the inheritance model that best fits the data, Akaike’s information criterion (AIC) was used. All data were adjusted for sex and age. The expected frequencies of genotypes were tested for accordance with Hardy–Weinberg equilibrium using χ2 tests. The significance threshold after implementation of the Bonferroni correction for multiple testing was *p* = 0.01. 

Statistical analysis was performed using StatTech v. 2.4.1 (Kazan, Russia) and GraphPad Prism 9.3.1 (San Diego, CA, USA). Categorical data were shown as absolute frequency (percentage) and continuous variables were expressed as mean  ±  SD or median (25th and 75th percentiles). Data normality was assessed using the Shapiro–Wilk test. Student’s *t*-test or ANOVA with Tukey post hoc tests were used to compare two or multiple groups, respectively, if quantitative parameters were distributed normally; otherwise, the non-parametric Mann–Whitney U-test or Kruskal–Wallis test with Dunn post hoc were applied. The differences in categorical parameters were assessed using the Pearson’s chi-square test or Fisher’s exact test. *p*-Values below 0.05 were considered significant. Stepwise multivariate linear regression analysis was performed to evaluate the effect of baseline predictors on changes of CRT after 3-IVI. Age, sex, SNP genotypes, and baseline OCT-markers were included in the multiple regression analysis.

## 3. Results

### 3.1. Association with nAMD Development

The genotypes of polymorphisms rs2285714 (CFI), rs10490924 (ARMS2), rs2230199 (C3), rs800292 (CFH), and rs6677604 (CFH) were determined in the case and in the control group (Table 3). For all these SNPs, genotype distribution was in accordance with the Hardy–Weinberg equilibrium in both groups.

For ARMS2 rs10490924, the G/T+T/T genotypes were associated with a 3.3-fold (OR = 3.35; CI: 2.06–5.45; *p* < 0.0001) and T/T with a 3.4-fold (OR = 3.42; CI: 1.93–6.05; *p* < 0.0001) increased odds of nAMD according to the dominant and recessive models, respectively. Each T allele increased the odds of developing nAMD by 2.4-fold under the additive model (OR = 2.43; CI: 1.76–3.36; *p* < 0.0001). According to AIC, the additive model was preferable.

The CFH rs800292 A/A+A/G genotypes were associated with 2.3-fold (OR = 0.44; CI: 0.28–0.69; *p* = 0.0004) decreased odds of nAMD according to the dominant model. Each A allele decreased the odds of developing nAMD by 2.0-fold in the additive model (OR = 0.49; CI: 0.33–0.72; *p* = 0.0002). The additive model was preferable.

According to the dominant model, the CFH rs6677604 A/A+A/G genotypes were associated with 2.8-fold (OR = 0.36; CI: 0.22–0.58; *p* < 0.0001) decreased odds of nAMD. Each A allele decreased the odds of developing nAMD by 2.4-fold in the additive model (OR = 0.41; CI: 0.27–0.63; *p* < 0.0001). The minimum AIC value was for the additive model.

No evidence of association with nAMD risk was observed for other SNPs (Table 4).

### 3.2. Association between SNPs and OCT-Markers

We evaluated 110 participants (115 eyes) who were treated with anti-VEGF therapy with 1-year follow-up. Baseline and dynamic OCT characteristics of study participants are shown in Table 5. According to the type of MNV by OCT data, MNV type 1 was diagnosed in 79 (68.7%) eyes and MNV type 2 in 36 (31.8%) eyes. The average number of injections was 6.7 ± 1.8. According to OCT, anti-VEGF therapy was highly effective in our study; a significant BCVA gain and a significant decrease of CRT, PED, and SRF height were achieved in all patients with complete resorption of the subretinal fluid in 107 (93%) eyes and complete adherence of the PED in 43 (37.4%) eyes to the end of follow-up (Table 5).

The effect of SNPs on the effectiveness of antiangiogenic therapy in patients with AMD was studied. The analysis showed that the baseline PED height depended on the rs2285714 CFI genotype (*p* = 0.018) (Table 6). In patients with C/T and T/T genotypes, the PED height was significantly higher than in carriers of the C/C variant. When comparing the group of C/T+T/T against the C/C genotype, the PED heights at baseline and after 3-IVI were significantly higher in carriers of the minor T allele than in the C/C genotype carriers (*p* = 0.005, *p* = 0.031, respectively). In addition, patients with the T allele had significantly higher CRT after three loading doses of IVI (*p* = 0.022, Table 7, dominant model).

The SRF height at baseline, and after 3-IVI significantly depended on the rs2285714 CFI genotype (*p* = 0.015, *p* = 0.007 respectively) and was higher in carriers of CT and TT genotypes (Table 6). When comparing the group of CT+TT against the CC genotype (dominant model), the SRF height at baseline and after 3-IVI was significantly higher in carriers of the minor T allele (*p* = 0.004, *p* = 0.002, respectively). However, the relationship was not detected in the recessive model (TT vs. CC+CT), most likely due to the small insufficient number of individuals with the TT genotype (Table 7).

By the end of one-year follow-up, the patients homozygous for the minor allele rs2285714 had significantly higher odds of active MNV (OR = 3.58, 95% CI: 1.05–12.28, *p* = 0.033) and presence of anastomoses and loops (OR = 3.21, 95% CI: 1.0–10.27, *p* = 0.041) (Table 7). Thus, in T/T carriers of the rs2285714 CFI genotype, recurrence of MNV was recorded significantly more frequently by the end of the follow-up. 

In patients with the protective allele rs800292 CFH, the SRF height after 3-IVI was lower than in patients with the risk G/G genotype (*p* = 0.04). Despite the low frequency of the protective A/A genotype rs800292 (CFH), it is worth noting that in all these patients, no IRCs were detected at baseline and there was no MNV activity after three loading doses of IVI.

Associations of rs2230199 with the baseline CRT and the IRC were revealed (Table 8). The baseline CRT was higher in carriers of the C allele of rs2230199 (*p* = 0.007). The patients homozygous for the G allele rs2230199 had significantly lower odds of IRC at baseline (OR = 0.280; 95% CI: 0.125–0.630) and after 3-IVI (OR = 0.296; 95% CI: 0.109–0.805).

No association between rs10490924 (ARMS2) and rs6677604 (CFH) SNPs and dynamics of OCT-markers was found (data not shown). In addition, we did not find a statistically significant pharmacogenetic relationship between SNPs and changes in visual acuity, nor did we find a statistically significant difference in the number of injections across different genotypes for any of the SNPs (data not shown).

Additionally, an associative analysis of polymorphisms with OCT markers of therapy effectiveness was performed using multivariate regression. The prognostic model characterizing the dependence of the BCVA and OCT parameter after three initial doses of IVI included variants of the genotypes of SNPs, type of MNV, the baseline BCVA, and the baseline OCT parameters (CRT, PED, SRF, IRC), with sex and age as predictors. 

Regression models for BCVA, PED, SRF, and IRC did not reveal significant associations with the studied polymorphisms. CRT after 3-IVI was significantly associated with baseline CRT and genotype of rs2285714. Table 9 summarizes the last step of the regression model. The resulting regression model was characterized by a correlation coefficient rxy = 0.744, which corresponds to a high correlation on the Chaddock scale. The model was statistically significant (*p* < 0.001). The resulting model explained 55.3% of the observed CRT 3-IVI variance. If all other variables were kept constant, for every 10 μm of CRT at baseline, an increase of approximately 5 μm in CRT 3-IVI would be expected (*p* < 0.001). According to the model, when changing the category of the CFI genotype to C/T, an increase in CRT 3-IVI by 20.9 μm would be expected, and when the category of the CFI genotype was changed to T/T, an increase in CRT 3-IVI by 24.0 μm would be expected. Under the dominant model, if all other variables were kept constant, eyes with the T allele would have an increase in CRT 3-IVI by 21.5 μm. Thus, the minor allele in CFI gene is associated with a smaller decrease in CRT. 

SRF height after 3-IVI was associated with type of MNV, SRF height at baseline, and presence of the T allele of rs2285714 under the dominant model. Table 10 summarizes the last step of the regression model. The resulting regression model was characterized by a correlation coefficient rxy = 0.717, which corresponds to a high correlation on the Chaddock scale. The model was statistically significant (*p* < 0.001). The resulting model explained 51.4% of the observed SRF 3-IVI variance. If all other variables were kept constant, eyes with MNV type 2 would have an SRF 3-IVI of 10.5 μm greater than eyes with MNV type 1, and for every 10 μm of SRF height at baseline, an increase of approximately 4 μm in SRF 3-IVI would be expected (*p* < 0.001). Under the dominant model, if all other variables were kept constant, eyes with the T allele would have an increase in SRF 3-IVI by 9.4 μm (Table 10).

### 3.3. Functional Annotation of the Studied Gene Polymorphisms

Among the five SNPs studied, three loci are missense variants and cause amino acid changes in the encoded polypeptides: rs10490924 ARMS2 (amino acid change Ala69Ser, SIFT score 0.00, SIFT prediction “damaging”), rs2230199 C3 (Arg102Gly, SIFT score 0.73, SIFT prediction “tolerated”), and rs800292 CFH (Val62Ile, SIFT score 1.00, SIFT prediction “tolerated”). In addition, the downstream gene variant (rs2285714 CFI) and intron variant (rs6677604 CFH) were determined. According to the SNPinfo and RegulomeDB databases, polymorphisms rs2230199 C3 and rs2285714 CFI have high regulatory potential. Notably, both SNPs were located in the regions of exonic splicing enhancer (ESE) and exonic splicing silencer (ESS). SNPs that are located at ESE or ESS may disrupt splicing activity and cause alternative splicing. 

According to the GTExportal database, all five SNPs are significantly included in the expression quantitative trait loci (eQTL). For example, rs2285714 has tissue-specific transcript associations with four genes (CASP6, MCUB, CFI, and PLA2GA12A) and is included in splicing quantitative trait loci (sQTL) with strong significance for CFI and PLA2G12A genes. It is important to note that the minor T allele of rs2285714 is associated with a dramatic decrease in the intron excision ratio for the CFI gene in 33 tissues, e.g., in the kidney cortex (NES (normalized effect size) = −1.2, *p*-value= 2.8 × 10^−10^), adipose tissue (NES = −1.1, *p*-value= 8.8 × 10^−71^), and nerve tissue (NES = −0.93, 4.4 × 10^−51^). The main results of in silico analysis are shown in Supplemental Table S1.

### 3.4. Effect of rs2285714 on Complement Factor I Plasma and Aqueous Humor Level

**Figure 1:**
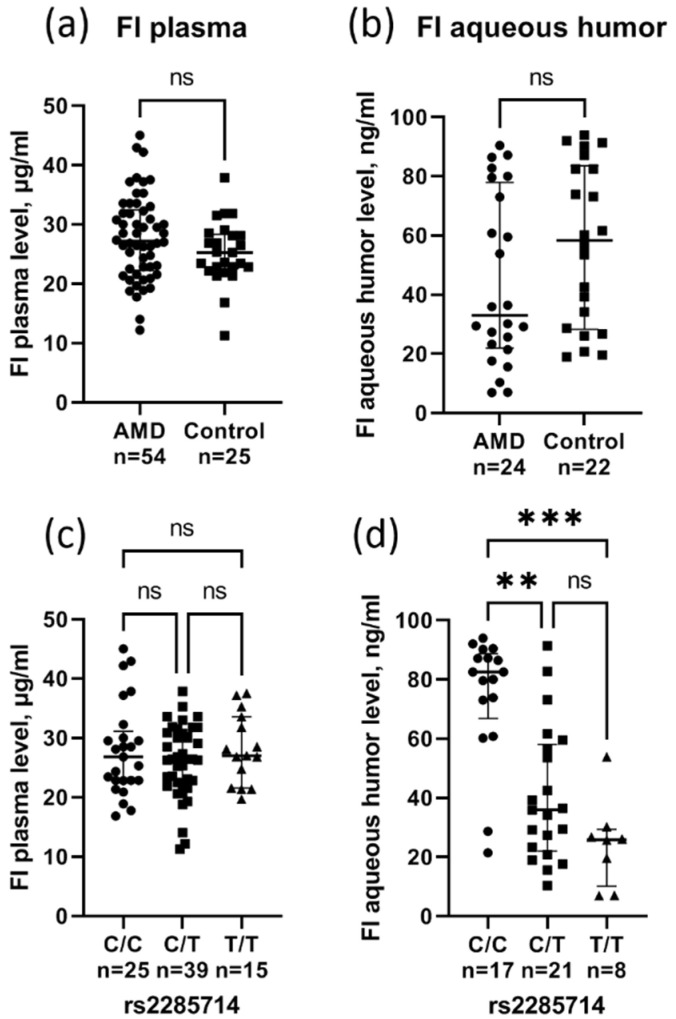
The complement factor I plasma (**a**) and aqueous humor (**b**) level in patients with AMD and controls (circles for AMD group, squares for control group). Effect of rs2285714 genotypes on FI levels in plasma (**c**) and aqueous humor (**d**) (circles for C/C group, squares for C/T group, triangles for T/T group). Median (q1–q3), ns: not significant, ** *p* < 0.01, *** *p* < 0.001 Kruskal–Wallis test with Dunn’s multiple comparisons test.

Complement factor I (FI) levels were measured by ELISA in plasma (54 AMD cases and 25 age-matched controls) and in aqueous humor (24 AMD cases and 22 age-matched controls). There was no difference in the FI plasma levels in AMD (27.8 ± 7.1 µg/mL) and control (25.3 ± 5.3 µg/mL) patients (Figure 1a). The median FI level in aqueous humor was lower in AMD patients (33.1 ng/mL) compared to controls (58.4 ng/mL), although the difference was not significant (*p* = 0.12) (Figure 1b). A huge concentration gradient between the plasma and aqueous humor was found. rs2285714 was significantly correlated with FI concentration in aqueous humor, but not in plasma (Figure 1c,d). The median FI level in aqueous humor was significantly higher in individuals with the C/C genotype (82.5 ng/mL) compared to individuals with the heterozygote (35.9 ng/mL, *p* = 0.002) and T/T genotypes (25.9 ng/mL, *p* = 0.0002).

## 4. Discussion

The complicated etiology of AMD has yet to be fully understood. Despite the fact that genetic studies have been highly successful in identifying genes and pathways driving AMD risk, our understanding of how these genetic variants contribute to AMD progression is still limited. [2]. Understanding the relationship of various genetic risks, the interactions with environmental factors, and causes of non-response to treatment may lead to new strategies for predicting and preventing disease progression [2]. 

As our results showed, patients with a minor allele for the *CFI* gene polymorphism are characterized by more severe macular lesions in nAMD and respond worse to antiangiogenic therapy. The T allele of rs2285714 has been associated with AMD disease severity, determined by OCT biomarkers (PED and SRF height at baseline). Importantly, *CFI* risk genotypes affect the disease response to treatment. Using regression analysis adjusted for baseline OCT biomarkers, we registered delayed and limited morphological responses (CRT and SRF height) after 3 monthly injections of aflibercept and higher odds of MNV recurrence at the end of follow-up in carriers of the minor allele of rs2285714. 

The CFI gene encodes the regulatory enzyme—serine protease FI—the function of which is to inhibit the alternative complement pathway. In the presence of a cofactor complement protein FH, the FI can cleave C3b deposited on the surface into inactive C3b, which cannot contribute to the complement amplification loop, and so the activity of FI might directly delay and inhibit complement activation [2]. Deregulation of the complement pathway has emerged as an important pathogenic factor in AMD [8,9,10]. Increased activation of the complement system is responsible for the chronic, low-level inflammation in tissue, or para-inflammation, that is characteristic of AMD. Inappropriate inflammatory responses have been linked to the pathogenesis of nAMD, and this might contribute to disease progression during anti-VEGF therapy treatment [18,23]. 

In the last decade a large number of studies has investigated associations of genetic polymorphisms with anti-VEGF treatment responses in nAMD. These pharmacogenetic studies were comprehensively reviewed in [3,19,24]. To date, association with outcomes after ranibizumab or bevacizumab treatment have been suggested for CFH (rs1061170, rs1065489, rs800292), ARMS2 (rs10490924), VEGFA (rs699947, rs699946, rs833069, rs3025000, rs943080, rs8330611), KDR (rs4576072, rs20715559), IL8 (rs4073), APOE (e4), FDZ4 (rs10898563), and others [3,19,24]. However, a number of studies has been unable to replicate these findings, including multicenter randomized trials (CATT and IVAN), which reported that no statistically significant association was found between genetic variants and anti-VEGF responsiveness [25,26]. 

The most consistent polymorphism associated with anti-VEGF treatment response is rs1061170 (CFH) [27,28,29]. It was found that patients with nAMD who had the minor allele of rs1061170 were at higher risk of responding poorly to anti-VEGF treatment and required additional anti-VEGF injections [29,30]. Abedi et al. [31] found the association of rs10490924 (A69S) in the ARMS2 gene with poor outcomes of intravitreal anti-VEGF injections in nAMD. A meta-analysis showed that A69S could be considered predictive of the anti-angiogenic effects, especially in Asian populations [32]. In another meta-analysis, anti-VEGF treatment was found to be more effective in patients homozygous for the VEGFA rs833061 minor allele [33]. A GWAS study including 919 Japanese patients showed the suggestive association of four SNPs relevant to the VEGF-related pathway (KCNMA1, SOCS2, and OTX2) with a lack of response to ranibizumab [34]. Our results are in line with the study of Wang et al. [35], who found rs2285714 association with anti-VEGF therapy response; the odds of having a TT or TC genotype among poor responders were three times greater than the odds of having the same genotypes among good responders. In addition, in a Turkish population, Aygun et al. found that RPE abnormalities were more frequent in the CT genotype of the rs2285714 polymorphism in dry-type AMD patients, and the mean subfoveal choroidal thickness was thinner in rs2285714 TT genotype carriers [36].

The following observations can be made from the analysis of the literature. First, genetic predisposition contributes to resistance to anti-VEGF therapy. Secondly, the finding of SNPs associated with response to anti-VEGF therapy is important for the early identification of nAMD patients who may benefit from alternative therapies such as drug switching. However, the genetic association with outcomes after anti-VEGF treatment in nAMD is still controversial. This is due to conflicting results and high heterogeneity in study designs: differences in treatment protocols, patient follow-up, intravitreal injection frequency, severity of the lesions at baseline, retreatment criteria, and visit scheduling, as well as variation in outcome measures. The heterogeneity in the results of these studies suggests that further research is needed to fully comprehend the influence of genotype on the anti-VEGF treatment [37]. In our opinion, the key role is played by the genetic traits of various populations. When a certain nucleotide influence on treatment response, the pharmacogenetic association could differ by ethnicity if the allele distribution of the candidate polymorphism varies among populations [38]. Therefore, the studies focused on the identification or replication of susceptibility genes in AMD development and in response to treatment in different populations do not lose their relevance.

From the beginning of treatment, the PED and SRF were worse in the T allele carriers of rs2285714. We can assume that this SNP somehow influences the faster progression of the disease. In an attempt to find the functional significance of polymorphic variants, we conducted an in silico analysis of their regulatory potential. rs2285714 refers to genetic variants that affect the splicing event (sQTL). sQTL is defined as genetic variants that are associated with changes in the splicing ratios of transcripts. According to the GTEX portal, rs2285714 is included in sQTL, which significantly affects the exon/intron excision ratio of the CFI gene, with evidence in 33 tissues. Genetic variants affecting splicing can have a stronger phenotypic impact than those affecting gene expression. Notably, sQTLs might actually contribute to complex traits and diseases at a similar or even larger degree than variants affecting gene expression [39]. 

The immunosorbent assay results showed that the effect of the CFI gene polymorphism on response to therapy was not associated with changes in the systemic level of FI; however, the content of FI in the aqueous humor was lower in patients with nAMD and in carriers of the minor allele of rs2285714. We suggest that the local production of FI, which affects the severity of the disease, may depend on the genotype of the CFI gene. Our results are in agreement with [40], which demonstrates increased complement activation in the aqueous humor of nAMD patients, supporting the hypothesis of a local dysregulation of the complement system in AMD. Another study [41] demonstrated that rare genetic CFI variants causing low FI levels are a substantial risk factor for AMD and identified several individual rare, type I CFI variants in patients with AMD with low FI levels. Currently it is accepted that the complement-mediated molecular mechanisms that drive AMD result from a combination of both locally synthesized complement proteins and systemic complement proteins that act locally in tissues [2]. It is not surprising that consecutive AMD disease stages showed increasing levels of complement activation, especially in individuals with hereditary load in complement genes [42]. Patients with AMD risk genetic variations may have greater background levels of inflammation, which may alter disease progression and likely contribute to a faster recurrence of neovascularization, resulting in a reduced treatment benefit [29]. Moreover, donor eyes with a hereditary risk at the CFH gene have significantly increased local C3b deposition, even before the manifestation of the disease [43].

Anti-VEGF therapy can only inhibit VEGF-induced neovascularization, but sustained activation of the complement system and inflammatory response may reduce the sensibility to anti-VEGF agents [18]. The increased inflammation may be found in patients with complement-related AMD-risk alleles, favoring the recurrence of neovascularization secondary to increased VEGF levels, and thus diminishing the response to anti-VEGF therapy in these patients [29]. MNV activity in nAMD is irregular, which is reflected in the range of the duration of dry intervals and late recurrences [20]. It can be cautiously assumed that the possible reasons for irregular MNV activity in nAMD are associated with the immune processes in the choroid. Moreover, recent study demonstrated dysregulation of the complement system following anti-VEGF therapy for nAMD [44]. The authors concluded that the interaction between anti-VEGF therapy and the complement system may be associated with ocular tissue damage after anti-VEGF treatment, affecting the clinical outcomes of anti-VEGF therapy. Regardless of whether it is pathogenic or not, VEGF production may return at some period. Nevertheless, prolonged unnecessary complement activation by anti-VEGF therapy might diminish the effectiveness of anti-VEGF injections or cause tissue injury due to local VEGF depletion [44]. In such cases, when determining a treatment regimen for patients with risk alleles in complement-associated genes, especially regulatory proteins (FI and FH), overtreatment should be taken into account to minimize the negative effects of anti-VEGF therapy. In this way personalized therapy has the potential to improve treatment outcomes. Individualized treatment is needed even more in developing countries due to the high cost of anti-VEGF therapy, in addition to the potential reduction of side effects for patients [45].

The limitation of our study is the relatively small sample size of the patient cohort, which decreases the statistical power available to identify statistically significant associations. In addition, one-year follow-up may be insufficient due to chronic disease duration. The advantage of this study is the inclusion of treatment-naive patients, which allowed us to eliminate the potential influence of the retreatment regimen. Our findings should be used to design larger confirmatory studies.

## 5. Conclusions

Our results suggest that the severity of AMD macular lesions is associated with rs2285714 and rs2230199 polymorphisms, which could be explained by their high regulatory potential. Patients with the minor allele of rs2285714 respond worse to antiangiogenic therapy. Unraveling the association between AMD genetics and response to therapy could pave the way for personalized AMD treatment, with genetic variants serving as predictors of its outcome, treatment regimen, and dosage mode.

## Figures and Tables

**Table 1:** SNPs analyzed in the study.

SNP	Location/Consequence	Chromosome Position/Orientation	Nucleotide Substitution	MAF
rs800292	Missense variant of the *CFH*: Val62Ile	1:196673103, plus	G>A	0.26
rs6677604	Intron variant of the *CFH*	1:196717788, plus	G>A	0.19
rs2285714	3′ untranslated region of *CFI*; synonymous variant of *PLA2G12A*	4:109717654, minus	C>T	0.39
rs10490924	Missense variant of *ARMS2*: Ala69Ser; 5′ untranslated region of *HTRA1*	10:122454932, plus	G>T	0.19
rs2230199	Missense variant of *C3*: Arg102Gly	19:6718376, minus	G>C	0.22

SNP: single nucleotide polymorphism. MAF: minor allele frequency in European populations according to 1000 Genomes.

**Table 2:** Primer/probe sequences.

rsID	Gene Name	Sequence
rs800292	*CFH*	Forward 5′-AAGGCACCCAGGCTATCTAT-3′
		Reverse 5′-TAATGGATTAAGAGCAACCCATTCT-3′
		5′-FAM-ATACCATTATT[+A][+T][+A]TTTCCAA-BHQ1-3′
		5′-VIC-CATACCATTATT[+A][+C][+A]TTTCCAA-BHQ1-3′
rs6677604	*CFH*	Forward 5′-ACACCAGAGCAGATACAGCA-3′
		Reverse 5′-TGCCACCAAAGCACAATACC-3′
		5′-FAM-CCTTTC[+C]T[+C]TCGCATTTTCTC-BHQ1-3′
		5′-VIC-CCTTTC[+C]C[+C]TCGCATTTTCTC-BHQ1-3′
rs2285714	*CFI/PLA2G12A*	Forward 5′-GTGTTTTCTGTACATCTCGGCA-3′
		Reverse 5′-TGCCTTTTGCAGCTTAACATTG-3′
		5′-FAM-TGCCACAGGTCTCATAGCACCTGT-BHQ1-3′
		5′-VIC-TGCCACAGGTTTCATAGCACCTGTC-BHQ2-3′
rs10490924	*ARMS2/HTRA1*	Forward 5′-AGTGACAAGCAGAGGAGCAA-3′
		Reverse 5′-CAGCAGGAGAGAAGAAGGCT-3′
		5′-FAM-CATGATCCCAGCTGCTAAAATCCA-BHQ1-3′
		5′-VIC-CCATGATCCCAGCTTCTAAAATCCAC-BHQ1-3′
rs2230199	*C3*	Forward 5′-TGGTCTTGTCTGTCTGGATGAA-3′
		Reverse 5′-CAAGATCCGGAAGCTGGAC-3′
		5′-FAM-CGAACTTGTTGCCCCCCTTTTC-BHQ1-3′
		5′-VIC-CGAACTTGTTGCGCCCCTTTT-BHQ1-3′

[+X]—LNA modifications.

**Table 3:** Allele and genotype frequencies in the nAMD and control groups in Western Siberia cohort.

SNP	Genotype/Allele	Case	Control	HWE ^a^ (Case)	HWE ^a^ (Control)
rs800292 G>A	G/G	138 (72%)	77 (52%)	0.79	0.84
	A/G	50 (26%)	60 (41%)		
	A/A	5 (3%)	10 (7%)		
	A	60 (16%)	80 (27%)		
rs6677604 G>A	G/G	152 (79%)	82 (56%)	0.3	0.67
	A/G	37 (19%)	54 (37%)		
	A/A	4 (2%)	11 (7%)		
	A	45 (12%)	76 (26%)		
rs2285714 C>T	C/C	61 (32%)	56 (38%)	1	0.39
	C/T	95 (49%)	65 (44%)		
	T/T	37 (19%)	26 (18%)		
	T	169 (44%)	117 (40%)		
rs10490924 G>T	G/G	40 (21%)	70 (48%)	0.31	0.26
	G/T	88 (46%)	58 (39%)		
	T/T	65 (34%)	19 (13%)		
	T	218 (56%)	168 (33%)		
rs2230199 G>C	G/G	121 (63%)	100 (68%)	0.51	0.57
	G/C	66 (34%)	41 (28%)		
	C/C	6 (3%)	6 (4%)		
	C	78 (20%)	53 (18%)		

^a^*p*-value for deviation of genotype distribution from the Hardy–Weinberg equilibrium (HWE).

**Table 4:** Association of the studied polymorphisms with the risk of nAMD in Western Siberia cohort.

SNP	Model of Inheritance	OR (95% CI) Adjusted for Sex and Age	*p*-Value	AIC
rs800292 G>A	Dominant: A/A+A/G vs. G/G	0.44 (0.28–0.69)	0.0004	453.7
	Recessive: A/A vs. G/G+A/G	0.36 (0.12–1.09	0.062	463
	Additive	0.49 (0.33–0.72)	0.0002	453
rs6677604 G>A	Dominant: A/A+A/G vs. G/G	0.36 (0.22–0.58)	<0.0001	448.5
	Recessive: A/A vs. G/G+A/G	0.27 (0.08–0.89)	0.022	461.2
	Additive	0.41 (0.27–0.63)	<0.0001	447.6
rs2285714 C>T	Dominant: C/T+T/T vs. C/C	1.36 (0.86–2.14)	0.19	464.8
	Recessive: T/T vs. C/C+C/T	1.12 (0.64–1.95)	0.7	466.4
	Additive	1.19 (0.87–1.61)	0.28	465.3
rs10490924 G>T	Dominant: G/T+T/T vs. G/G	3.35 (2.06–5.45)	<0.0001	441.7
	Recessive: T/T vs. G/T+G/G	3.42 (1.93–6.05)	<0.0001	446.6
	Additive	2.43 (1.76–3.36)	<0.0001	434.1
rs2230199 G>C	Dominant: C/G+G/G vs. G/G	1.33 (0.84–2.11)	0.22	465
	Recessive: C/C vs. G/G+C/G	0.82 (0.25–2.61)	0.73	466.4
	Additive	1.21 (0.81–1.79)	0.35	465.6

CI: confidence interval; OR: odds ratio; SNP: single nucleotide polymorphism. AIC: Akaike’s information criterion. Since overall five SNPs were tested, the significance threshold after implementation of Bonferroni correction for multiple testing is *p* = 0.01.

**Table 5:** Characterization of functional and anatomical parameters of the retina in patients with nAMD at baseline and during anti-VEGF therapy.

	Baseline	3-IVI	5-IVI	Final	*p*-Value
BCVA, letters	46 ± 22	54 ± 21	69 ± 14	70 ± 14	<0.001 ^a^
CRT, μm	341 ± 69	265 (234–306)	234 (200–284)	211 (190–262)	<0.001 ^b^
PED, abs. %	115 (100)	95 (82.6)	77 (66.9)	72 (62.6)	
PED height, μm	123 (89–167)	45 (23–78)	44 (23–63)	32 (21–61)	<0.001 ^b^
SRF, abs. %	107 (93)	80 (69.6)	33 (28.7)	8 (7)	
SRF height, μm	67 (34–102)	34 (23–56)	22 (12–29)	19 ± 8	<0.001 ^b^
IRF, abs. (%)	89 (77.4)	59 (51.3)	21 (18.3)	4 (3.5)	<0.001 ^c^
IRC, abs. (%)	52 (45.2)	20 (17.7)	-	-	<0.001 ^d^

BCVA—best corrected visual acuity; CRT—central retinal thickness; PED—detachment of the pigment epithelium; SRF—subretinal fluid; IRC—intraretinal cysts; IRF—intraretinal fluid. Mean ± SD or Median [q1–q3]. Applied methods for matched samples: ^a^ repeated measures ANOVA, ^b^ Friedman test, ^c^ Cochran’s Q-test, ^d^ McNemar test.

**Table 6:** Analysis of the OCT biomarkers depending on the rs2285714 genotype.

Parameter		rs2285714			*p*-Value
		C/C	C/T	T/T	
PED baseline, μm		111 (74–127)	126 (106–182)	145 (100–167)	**0.018 ^a^** **p_C/T-C/C_ = 0.025**
PED 3-IVI, μm		30 (2–45)	42 (23–78)	45 (23–67)	0.093 ^a^
PED 5-IVI, μm		23 (0–35)	28 (0–56)	32 (0–45)	0.265 ^a^
PED final, μm		16 (0–33)	21 (0–48)	11 (0–32)	0.549 ^a^
SRF baseline, μm		46 (23–75)	79 (41–118)	67 (47–90)	**0.015 ^a^** **p_C/T-C/C_ = 0.014**
SRF 3-IVI, μm		11 (0–26)	32 (10–56)	34 (11–56)	**0.007 ^a^** **p_C/T-C/C_ = 0.007**
SRF 5-IVI	Absence (%)	34 (85.0)	36 (64.3)	10 (58.8)	**0.044 ^b^**
	Presence (%)	6 (15.0)	20 (35.7)	7 (41.2)	
SRF final	Absence (%)	39 (97.5)	52 (92.9)	14 (82.4)	0.125 ^b^
	Presence (%)	1 (2.5)	4 (7.1)	3 (17.6)	
CRT baseline, μm		340 (288–381)	335 (292–382)	340 (288–394)	0.688 ^a^
CRT 3-IVI, μm		241 (220–294)	270 (246–310)	270 (259–335)	0.051 ^a^
CRT 5-IVI, μm		228 (200–278)	244 (221–282)	245 (210–300)	0.152 ^a^
CRT final, μm		201 (190–259)	212 (200–258)	230 (198–279)	0.180 ^a^
IRC initial	Absence (%)	22 (55.0)	31 (55.4)	9 (52.9)	0.985 ^b^
	Presence (%)	18 (45.0)	25 (44.6)	8 (47.1)	
IRC 3-IVI	Absence (%)	32 (84.2)	45 (80.4)	14 (82.4)	0.892 ^b^
	Presence (%)	6 (15.8)	11 (19.6)	3 (17.6)	
Anastomoses and loops final	Absence (%)	32 (86.5)	43 (82.7)	10 (62.5)	0.112 ^b^
	Presence (%)	5 (13.5)	9 (17.3)	6 (37.5)	
Active MNV final	Absence (%)	35 (87.5)	51 (91.1)	12 (70.6)	0.091 ^b^
	Presence (%)	5 (12.5)	5 (8.9)	5 (29.4)	

Applied method: ^a^ Kruskal–Wallis test and Dunn’s criterion with Holm correction as a post hoc method, ^b^ Pearson’s chi-square test.

**Table 7:** Analysis of the OCT biomarkers depending on the rs2285714 (C/C vs. C/T+T/T) T allele presence.

Parameter		rs2285714 (Dominant Model)		*p*-Value
		C/C	C/T+T/T	
PED baseline, μm		111 (74–127)	134 (100–179)	**0.005 ^a^**
PED 3-IVI, μm		30 (2–45)	44 (23–78)	**0.031 ^a^**
PED 5-IVI, μm		23 (0–35)	32 (0–56)	0.144 ^a^
PED final, μm		16 (0–33)	21 (0–46)	0.427 ^a^
SRF baseline, μm		46 (23–75)	78 (41–114)	**0.004 ^a^**
SRF 3-IVI, μm		11 (0–26)	34 (11–56)	**0.002 ^a^**
SRF 5-IVI	Absence (%)	34 (85.0)	46 (63.0)	**0.014 ^b^**
	Presence (%)	6 (15.0)	27 (37.0)	
SRF final	Absence (%)	39 (97.5)	66 (90.4)	0.256 ^b^
	Presence (%)	1 (2.5)	7 (9.6)	
CRT baseline, μm		339 ± 77	343 ± 66	0.770 ^c^
CRT 3-IVI, μm		241 (220–294)	270 (247–312)	**0.022 ^a^**
CRT 5-IVI, μm		228 (200–278)	245 (220–290)	0.053 ^a^
CRT final, μm		201 (190–259)	220 (200–263)	0.087 ^a^
IRC initial	Absence (%)	22 (55.0)	40 (54.8)	0.983 ^b^
	Presence (%)	18 (45.0)	33 (45.2)	
IRC 3-IVI	Absence (%)	32 (84.2)	59 (80.8)	0.659 ^b^
	Presence (%)	6 (15.8)	14 (19.2)	
Anastomoses and loops final	Absence (%)	32 (86.5)	53 (77.9)	0.287 ^b^
	Presence (%)	5 (13.5)	15 (22.1)	
Active MNV final	Absence (%)	35 (87.5)	63 (86.3)	1.000 ^b^
	Presence (%)	5 (12.5)	10 (13.7)	
		**rs2285714 (recessive model)**		
		**C/C+C/T**	**T/T**	
PED baseline, μm		123 (89–168)	145 (100–167)	0.311 ^a^
PED 3-IVI, μm		34 (19–78)	45 (23–67)	0.690 ^a^
PED 5-IVI, μm		23 (0–55)	32 (0–45)	0.816 ^a^
PED final, μm		20 (0–44)	11 (0–32)	0.639 ^a^
SRF baseline, μm		63 (33–96)	67 (47–90)	0.531 ^a^
SRF 3-IVI, μm		23 (0–45)	34 (11–56)	0.395 ^a^
SRF 5-IVI	Absence (%)	70 (72.9)	10 (58.8)	0.239 ^b^
	Presence (%)	26 (27.1)	7 (41.2)	
SRF final	Absence (%)	91 (94.8)	14 (82.4)	0.099 ^b^
	Presence (%)	5 (5.2)	3 (17.6)	
CRT baseline, μm		337 (289–381)	340 (288–394)	0.388 ^a^
CRT 3-IVI, μm		264 (233–302)	270 (259–335)	0.133 ^a^
CRT 5-IVI, μm		234 (201–280)	245 (210–300)	0.502 ^a^
CRT final, μm		211 (190–258)	230 (198–279)	0.229 ^a^
IRC initial	Absence (%)	53 (55.2)	9 (52.9)	1.000 ^b^
	Presence (%)	43 (44.8)	8 (47.1)	
IRC 3-IVI	Absence (%)	77 (81.9)	14 (82.4)	0.966 ^b^
	Presence (%)	17 (18.1)	3 (17.6)	
Anastomoses and loops final	Absence (%)	75 (84.3)	10 (62.5)	**0.041 ^b^**
	Presence (%)	14 (15.7)	6 (37.5)	
Active MNV final	Absence (%)	86 (89.6)	12 (70.6)	**0.033 ^b^**
	Presence (%)	10 (10.4)	5 (29.4)	

Applied method: ^a^ Mann–Whitney U-test, ^b^ Pearson’s chi-square test, ^c^ Student’s *t*-test.

**Table 8:** Analysis of the OCT biomarkers depending on the rs2230199.

Parameter		rs2230199 (Dominant Model)		*p*
		C/C+C/G	G/G	
PED baseline, μm		123 (89–171)	123 (99–167)	0.705 ^a^
PED 3-IVI, μm		34 (11–62)	45 (22–78)	0.373 ^a^
PED 5-IVI, μm		23 (0–45)	30 (0–56)	0.793 ^a^
PED final, μm		18 (0–33)	20 (0–45)	0.875 ^a^
SRF baseline, μm		67 (40–90)	63 (29–109)	0.616 ^a^
SRF 3-IVI, μm		23 (0–42)	23 (0–55)	0.510 ^a^
SRF 5-IVI	Absence (%)	28 (70.0)	52 (71.2)	0.890 ^b^
	Presence (%)	12 (30.0)	21 (28.8)	
SRF final	Absence (%)	36 (90.0)	69 (94.5)	0.451 ^b^
	Presence (%)	4 (10.0)	4 (5.5)	
CRT baseline, μm		336 (273–405)	283 (227–345)	**0.007 ^a^**
CRT 3-IVI, μm		282 ± 51	269 ± 53	0.220 ^c^
CRT 5-IVI, μm		234 (208–288)	234 (201–280)	0.683 ^a^
CRT final, μm		211 (200–270)	211 (190–256)	0.791 ^a^
IRC initial	Absence (%)	14 (35.0%)	48 (65.8)	**0.002 ^b^**
	Presence (%)	26 (65.0)	25 (34.2)	
IRC 3-IVI	Absence (%)	28 (70.0)	63 (88.7)	**0.014 ^b^**
	Presence (%)	12 (30.0)	8 (11.3)	
Anastomoses and loops final	Absence (%)	31 (77.5)	54 (83.1)	0.480 ^b^
	Presence (%)	9 (22.5)	11 (16.9)	
MNV final	Absence (%)	33 (82.5)	65 (89.0)	0.389 ^b^
	Presence (%)	7 (17.5)	8 (11.0)	

Applied method: ^a^ Mann–Whitney U-test, ^b^ Pearson’s chi-square test, ^c^ Student’s *t*-test.

**Table 9:** Final step of stepwise linear regression showing significant predictors of CRT after 3-IVI.

	B Coefficient	Standard Error	*p*-Value
CRT baseline	0.533	0.049	<0.001
rs2285714: C/T	20.867	7.516	0.007
rs2285714: T/T	24.001	10.652	0.026
rs2285714: C/T+T/T (vs. C/C)	21.570	7.131	0.003

**Table 10:** Final step of stepwise linear regression showing significant predictors of SRF height after 3-IVI.

	B Coefficient	Standard Error	*p*-Value
MNV: type 2	10.480	4.635	0.026
SRF height baseline	0.412	0.046	<0.001
rs2285714: C/T+T/T (vs. C/C)	9.440	4.658	0.045

## Data Availability

The datasets are available from the corresponding author upon request.

## Supplementary Materials

The following supporting information can be downloaded at: www.mdpi.com/article/10.3390/biomedicines10071658/s1, Figure S1: Allelic discrimination and Sanger verification results; Table S1: Regulatory effects of the SNPs selected for the study and their effect on gene expression.
