# Supplementary material for: Association between Polymorphisms in CFH, ARMS2, CFI, and C3 Genes and Response to Anti-VEGF Treatment in Neovascular Age-Related Macular Degeneration
Source: Biomedicines. 2022 Jul 10;10(7):1658. doi: 10.3390/biomedicines10071658 (PMC9312436; doi:10.3390/biomedicines10071658)
Supplement: Supplementary file 1 [file biomedicines-10-01658-s001.zip › biomedicines-1723750-supplementary.pdf]

Table S1. Regulatory effects of the SNPs selected for the study and their effect on gene expression

SNPs	Regulatory effects according to HaploReg, SNPinfo, Regulome DB, VannoPortal										Expression QTLs (GTEx) genes/ tissues	Splicing QTLs (GTEx) genes/ tissues
	Prom histone marks	Enh histone marks	DNAse	Altered reg motifs	TF Binding evidence	Rank / Score RegulomeDB	Splicing ESE/ ESS	SIFT/ CADD	Reg Score Vanno Portal	Reg Potential SNPinfo		
rs10490924 *ARMS2* missense	-	-	**yes**	15	5	5/0,13	–	Damaging	0.192	–	ARMS2: 8<br>PLEKHA1: 6<br>HTRA1: 1	ARMS2: 1<br>PLEKHA1: 1
rs2230199 *C3* missense	**yes**	**yes**	**yes**	28	29	**3a/0,86**	**yes**	Tolerated	**0.584**	**0,33**	GPR108: 7<br>CTD-3128G10.7: 1	-
rs2285714 *CFI/ PLA2G12A* downstream variant	-	-	-	10	7	**4/0,60**	**yes**	Likely pathogenic	0.327	**0,36**	CFI: 1<br>PLA2G12A:23<br>MCUB:1<br>CASP6: 4	CFI: 33<br>PLA2G12A: 7
rs6677604 *CFH* intron variant	-	**yes**	**yes**	20	16	5/0,13	–	–	0.315	–	CFHR3: 27<br>CFHR1: 18<br>CFHR4: 1<br>ZBTB41: 1	CFH: 12<br>CFHR1: 3
rs800292 *CFH* missense	-	-	-	10	-	7/0,18	–	Tolerated/	0.156	0,26	CFH: 8<br>KCNT: 5<br>CFHR3: 5<br>CFHR1: 1<br>CFHR4: 1	CFH: 3<br>CFHR2: 1<br>CFHR1: 1<br>F13B: 1

CADD - Combined Annotation Dependent Depletion tool; DNAse – DNAse hypersensitive regions; Enh – Enhancer; ESE - Exonic Splicing Enhancer, ESS – Exonic Splicing Silencer; Prom – Promoter; Reg – regulatory; TF - Transcript Factor.

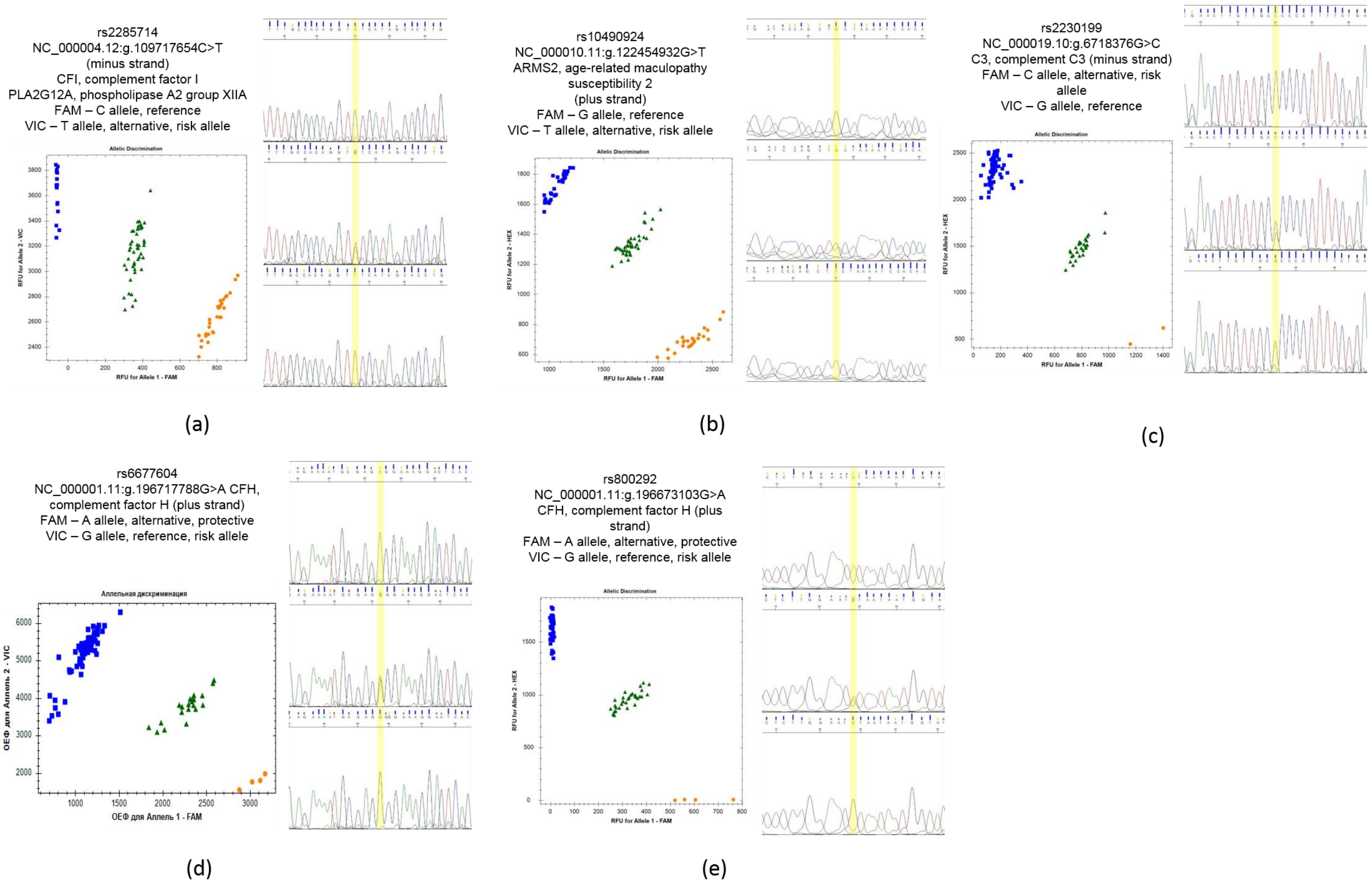

Figure S1. Allelic discrimination and Sanger verification results of rs2285714 (a), rs10490924 (b), rs2230199 (c), rs6677604 (d), rs800292 (e).
